# Effect of seed removal by ants on the host-epiphyte associations in a tropical dry forest of central Mexico

**DOI:** 10.1093/aobpla/ply056

**Published:** 2018-09-25

**Authors:** Carmen Agglael Vergara-Torres, Angélica Ma Corona-López, Cecilia Díaz-Castelazo, Víctor Hugo Toledo-Hernández, Alejandro Flores-Palacios

**Affiliations:** 1 Centro de Investigación en Biodiversidad y Conservación (CIByC), Universidad Autónoma del Estado de Morelos, Av. Universidad, Col. Chamilpa, Cuernavaca, Morelos, México; 2 Red Ecología Funcional, Instituto de Ecología, A. C. Carretera Antigua a Coatepec, El Haya, Xalapa, Veracruz, México

**Keywords:** Host limitation, host preference, plant–ant interaction, plant–plant interaction, seed depredation, *Tillandsia*

## Abstract

Seed depredation is recognized as a determining factor in plant community structure and composition. Ants are primary consumers of seeds influencing abundance of epiphytes on trees. This study was conducted in two subunits of a tropical dry forest established on different soil substrates in San Andrés de la Cal, Tepoztlán, in Morelos, Mexico, and experimentally tested whether seed removal activity is higher in tree species with smaller epiphyte loads compared to those with greater epiphyte loads. Five trees were selected at random from six species of trees with high (preferred hosts) or low (limiting hosts) epiphyte loads. Seed removal differed among hosts and different soil substrates in the forest. On relating seed removal to the abundance of arboreal ants, the most consistent pattern was that lower seed removal was related to lower ant abundance, while high seed removal was associated with intermediate to high ant abundance. Epiphyte seed removal by ants influences epiphyte abundance and can contribute considerably to a failure to establish, since it diminishes the quantity of seeds available for germination and establishment.

## Teaser

We found evidence that host preference by epiphytes could be highly influenced by herbivores, as herbivores can reduce the amount of seeds that arrive on the trees.

## Introduction

The forest canopy consists of tree crowns, including foliage, branches and epiphytes, and is a zone of interface between the processes of the atmosphere and those of the soil (Nadkarni 1995; Shaw 2004). The canopy hosts a great richness of species and varieties of life forms, including epiphytic plants, which grow on the structure of other plants. For at least one part of their life cycle, the roots of these epiphytic plants are not in contact with the soil and they never develop haustoria (Flores-Palacios 2016). The presence of epiphytic flora increases the species richness and structural complexity of the forest, as well as providing resources for fauna and offering new sources and routes for nutrient and water cycling in the forest (Coxson and Nadkarni 1995; Cruz-Angón and Greenberg 2005).

Vascular epiphytes account for around 9 % of vascular plants worldwide (Zotz 2013). At the regional scale, the abundance, richness and composition of epiphytic species in a forest depend on the trees themselves (Vergara-Torres *et al.* 2010). Some studies in tropical dry forests have found that epiphytic plant species abundance differs among the trees of a forest. These differences may be due to differences in bark texture, chemical content and seed adherence among phorophytes (Valencia-Díaz *et al.* 2010; Vergara-Torres *et al.* 2010; Victoriano-Romero *et al.* 2017). It has been hypothesized that anemochorous seed dispersion in epiphytes allows them to colonize all potential hosts (Callaway *et al.* 2002), but the empirical evidence shows that such generalized colonization of trees is more likely to be an exception (Flores-Palacios *et al.* 2015b). What therefore are the factors that limit epiphytes to one group of hosts, considering that some species can even live on electricity cables (Abril and Bucher 2009; Wester and Zotz 2010)?

The particularly diverse richness of arthropods that exists in the canopy of tropical forests affects different trophic levels of the ecosystem because of the functions these organisms perform as predators, pollinators, detritivores or parasites (Basset *et al.* 2003; Ozanne *et al.* 2003). Ant (Hymenoptera: Formicidae) species have colonized all of the habitat types in the forests (from the soil to the tree crowns). Their success lies in the diversity of ecological niches and associations they have developed with other organisms, their influence over other species and their role as ecosystem engineers (Folgarait 1998; Longino *et al.* 2002). It has been reported that a single tree in a tropical forest can host >30 ant species (Tobin 1995; Schulz and Wagner 2002). Recognized associations, specifically between ants and epiphytic plants, include depredation, opportunistic occupation and mutualism (e.g. ant gardens, myrmecophytic epiphytes) (Rico-Gray and Oliveira 2007). In addition to being considered primary consumers of seeds (Morton 1985; Johnson 2001; Beattie and Hughes 2002), ants may also act as primary and secondary agents of dispersion, since they collect seeds that have fallen from the trees or have been dispersed by the wind or in the faeces of animals (Levey and Byrne 1993; Peters *et al.* 2003).

One well-studied factor is the impact of seed depredation on the population dynamics of plants (e.g. *Silphium integrifolium*, Howe and Brown 2000; *Lupinus* and *Lithospermum*, Bricker *et al.* 2010; *Eucalyptus miniata*, Setterfield and Andersen 2018). Seed depredation has been recognized as a determining factor in the structure and composition of plant communities. For example, ants can reduce the amount of seed available for germination, consequently reducing the number of seedlings (e.g. *Taxus baccata* in a Mediterranean forest, Hulme 1997; *Secale cereale* in a coniferous forest and mesic steppe, Connolly *et al.* 2014; *Pentaclethra macrophylla* in a lowland forest, Rosin and Poulsen 2016). Associations between trees and ants could explain epiphyte removal if the ants clean the trees of structural parasites, as has been suggested for tropical forest trees (Janzen 1971), such that a mutualist activity with the trees triggers an amensalist interaction with the epiphytes. However, there has been a particular lack of field experimentation in which epiphytes could be manipulated to examine the different types of interactions that occur with their dispersal agents or with those that act to limit establishment once the seeds have become anchored to a host.

This study was carried out in two contiguous subunits of a tropical dry forest differing in the parent soil substrate and as a consequence in flora and ant compositions. We conducted experiments on six host species with different abundances of epiphytic plants (preferred and limiting), selected according to previous observations (Vergara-Torres *et al.* 2010; Cortés-Anzúres 2015) to determine the extent to which ants associated with the trees can remove the seeds of four epiphytic plant species. In two subunits of a forest with different compositions of trees, ants and epiphytes, we experimentally tested the hypothesis that the activity of seed removal by the ants will be greater in tree species with lower epiphyte loads than in those with higher loads.

## Methods

### Study area

The study was conducted in the tropical dry forest of the ejido of San Andrés de la Cal, Tepoztlán, in Morelos state, central Mexico (18°57ʹ22.2ʺN–99°06ʹ50.2ʺW; 1480–1670 m a.s.l.). The climate is semi-warm sub-humid, accordingly to the Köppen climatic system modified for Mexico follows the formula (A) Cw2(w)ig, with an annual mean temperature >18 °C and mean annual precipitation of between 800 and 1000 mm (Comision Nacional del Agua, unpubl. data). In the study area, the tropical dry forest develops two subunits (*sensu*Mueller-Dombois and Ellenberg 1974) on two different soil types: one subunit over limestone rock and the other over volcanic rock but the species that form the community of trees and epiphytes, and the epiphyte-host associations, are known in both of these subunits (Vergara-Torres *et al.* 2010; Cortés-Anzúres 2015). Nineteen species of epiphytes have been recorded in the limestone forest and 15 species of epiphytes are known in the lava spill forest (Vergara-Torres *et al.* 2010; Cortés-Anzúres 2015); however, the abundance of species differs (Table 1). In the limestone forest, the dominant woody species include *Sapium macrocarpum*, *Bursera fagaroides*, *B. glabrifolia* and *Conzattia multiflora* (Table 2). The distribution of epiphyte species is not random among the trees, since *Bursera copallifera*, *B. glabrifolia* and *B. bipinnata* support more epiphyte individuals than would be expected by chance and have thus been considered preferred hosts; while *C. multiflora*, *Ipomoea murucoides* and *I. pauciflora* are limiting hosts, since these present a low abundance of epiphytes (Vergara-Torres *et al.* 2010). Other hosts, such as *S. macrocarpum* and *Bunchosia canescens* are indefinite hosts, i.e. they do not demonstrate a clear pattern of preference for or limitation of the epiphytic plants. However, the tendency in *S. macrocarpum* was towards a lower abundance of epiphytes (Vergara-Torres *et al.* 2010).

**Table 1.** Species of epiphytic bromeliads that inhabit the woody plants (DBH > 3 cm) in two forest subunits (limestone and lava flow) of the tropical dry forest of San Andrés de la Cal, Tepoztlán, in Morelos, Mexico. The percentage of individuals found in each forest subunit is shown for each epiphyte species (Vergara-Torres *et al.* 2010; Cortés-Anzúres 2015).

True epiphytes	Limestone	Lava flow
*Tillandsia achyrostachys*	12.4 %	–
*Tillandsia caput-medusae*	2.5 %	2.0 %
*Tillandsia cryptantha*	–	0.2 %
*Tillandsia circinnatioides*	0.2 %	–
*Tillandsia hubertiana*	2.7 %	20.0 %
*Tillandsia ionantha*	0.1 %	0.1 %
*Tillandsia makoyana*	0.2 %	0.2 %
*Tillandsia recurvata*	76.7 %	2.5 %
*Tillandsia schiedeana*	2.4 %	75.0 %
*Viridantha atroviridipetala*	2.8 %	–

**Table 2.** The most abundant tree species found in two forest subunits (limestone and lava flow) of the tropical dry forest of San Andrés de la Cal, Tepoztlán, in Morelos, Mexico. The percentage of the total number of individuals in each forest subunit is shown.

Tree species	Limestone	Lava flow
Apocynaceae		
*Plumeria rubra*	0.7 %	3.0 %
*Thevetia thevetioides*	2.6 %	1.3 %
Burseraceae		
*Bursera bicolor*	1.4 %	–
*B. bipinnata*	2.4 %	2.6 %
*B. copallifera*	2.6 %	1.3 %
*B. fagaroides*	14.9 %	1.7 %
*B. glabrifolia*	11.1 %	0.4 %
Convolvulaceae		
*Ipomoea murucoides*	5.5 %	2.1 %
*I. pauciflora*	9.6 %	8.2 %
Euphorbiaceae		
*Sapium macrocarpum*	19.0 %	32.2 %
Fabaceae		
*Conzattia multiflora*	6.3 %	–
*Lysiloma acapulcense*	2.4 %	4.3 %
Fagaceae		
*Quercus obtusata*	–	6.4 %
Lamiaceae		
*Salvia sessei*	–	4.7 %
Malpighiaceae		
*Bunchosia canescens*	4.3 %	–
Meliaceae		
*Cedrela odorata*	–	3.0 %
*Trichilia hirta*	2.0 %	0.4 %
Oleaceae		
*Fraxinus uhdei*	–	3.9 %
Rutaceae		
*Zanthoxylum fagara*	–	3.0 %
Tiliaceae		
*Heliocarpus terebinthinaceus*	2.0 %	0.9 %

Other species	13.2 %	20.6 %

In the lava-flow forest, the epiphytic plants are distributed among 32 tree species (Table 2), the most abundant of which are *S. macrocarpum*, *I. pauciflora* and *Quercus obtusata*. The species *B. bipinnata* and *Q. obtusata* are preferred hosts, while *S. macrocarpum*, *I. pauciflora*, *Salvia sessei* and *I. murucoides* are limiting hosts (Cortés-Anzúres 2015).

In this forest, 27 ant species have been recorded; 19 in the limestone subunit and 17 in the lava-flow subunit, with only nine species of ants shared across both forest subunits (Vergara-Torres *et al.* 2017). The dominant ant species in the trees of the forest are *Camponotus rectangularis willowsi* and *Crematogaster curvispinosa*. These dominant species are the only species previously recorded in all of the species of trees used in the present study (Vergara-Torres *et al.* 2017).

### Removal experiment

In the study area, seed dispersion of *Tillandsia* only occurs during the end of the dry season (April–May; Victoriano-Romero *et al.* 2017). In order to conduct the removal experiment, mature inflorescence capsules of *Tillandsia caput-medusae*, *T. hubertiana*, *T. schiedeana* and *T. recurvata* were collected in the months of February and March, which is when the fruits mature and begin to open (Flores-Palacios *et al.* 2015a). The capsules were transported to the laboratory and placed in a drying oven (Binder, model FD 115-UL, USA) in order to complete drying and opening. Once the seeds were released, they were divided into lots of 30 seeds each. Each lot per species was inserted into the strands of a cotton thread. In total, 240 lots were produced, 60 per epiphyte species. Five individuals each of six tree species were selected in the two forest subunits (limestone and lava flow) on which to place the seed lots. The selected species were *B. bipinnata* and *B. copallifera*, species of trees preferred by the epiphytes, and *B. fagaroides*, *I. murucoides*, *I. pauciflora* and *S. macrocarpum*, which in this study were considered limiting species for the epiphytes (Table 1). Fieldwork was conducted from May to August 2014, during the rainy season, when *Bursera* species and *S. macrocarpum* flowering occurs (C. A. Vergara-Torres, pers. observ.).

Eight branches of <4 cm in diameter were selected on each tree. One thread containing one seed lot of each epiphyte species was assigned at random and attached to each branch. Four of the lots were protected from the actions of herbivores, using a tanglefoot barrier (Tanglefoot Company, product number 99080) placed on the base of the branch. This barrier consisted of a ring with a resin that goes around the entire circumference of the branch and functioned as an obstacle to ambulant insects. Seed lots were also placed on another four branches without a tanglefoot barrier, such that insects could pass freely. All seed lots were reviewed once per week and the number of removed seeds recorded.

The abundance of the dominant ant species (*C. r. willowsi* and *C. curvispinosa*; Vergara-Torres *et al.* 2017) was recorded in the canopy of the study zone. On the same trees that contained the experimental lots, weekly daytime samples were taken. The trees were climbed using a ladder and mountain climbing equipment and ants were collected using a paint brush moistened with 70 % ethylic alcohol and/or an entomological aspirator, searching directly on the branches of the trees.

### Data analysis

In order to determine whether the proportion of depredated seeds in the lots without a tanglefoot barrier differed between preferred and limiting hosts, a generalized linear model (GLM) for a binomial distribution was conducted, using a logit link (Crawley 1993). Seed lots protected with tanglefoot were excluded from this analysis since seed removal was very low in these lots. This also applied to the tree species *B. bipinnata* and *I. pauciflora* in the lots of *T. caput-medusae*, *T. hubertiana* and *T. schiedeana*, where no removal was recorded (see Results). All analysis was conducted using the program Stata 13 (StataCorp 2013).

With a non-parametric Spearman rank correlation analysis (Zar 2010), we explored the relationship between the proportion of removed seeds and ant abundance among the host and forest subunits. In order to conduct this analysis, we used the proportional abundance of *C. r. willowsi* and *C. curvispinosa* reported previously between these host trees (Vergara-Torres *et al.* 2017), as well as the mean proportion of removed seeds per host species observed during our experiment.

## Results

No removal occurred from the protected lots (Table 3), except for two lots of seeds of *T. caput-medusae* and *T. schiedeana* on the host *I. murucoides*. However, this incidence of seed removal occurred because the branches and leaves of this tree created bridges over the tanglefoot barrier, providing an access route to the seeds. Due to the lack of removal from the protected lots, this treatment was not included in the analysis. There was no accidental fall of the seeds from the thread, which suggests that the seeds that disappeared were removed by the ants that forage on the branches and were consistently found trapped on the tanglefoot barrier.

**Table 3.** Percentage of germinated, non-germinated and removed seeds in seed lots of four *Tillandsia* species attached to six tree species with two treatments; (i) protected from the actions of herbivores with a tanglefoot barrier (P), and (ii) not protected (NP). The percentages are of the total number of seeds placed on five individual trees per tree species.

Epiphyte species	Tree species	*N*	Percentage of germinated seeds		Percentage of non-germinated seeds		Percentage of depredated seeds	
			P	NP	P	NP	P	NP
*T. caput-medusae*	*Bursera bipinnata*	150	95	77	5	11		12
	*B. copalloifera*	150	79	57	21	10		33
	*B. fagaroides*	150	93	68	7	4		28
	*Ipomoea murucoides*	150	58	76	42	4		20
	*I. pauciflora*	150	99	96	1	4		0
	*Sapium macrocarpum*	150	89	81	11	7		12
*T. hubertiana*	*B. bipinnata*	150	53	81	47	7		12
	*B. copalloifera*	150	57	77	43	10		13
	*B. fagaroides*	150	100	53	0	5		43
	*I. murucoides*	150	99	57	1	23		20
	*I. pauciflora*	150	83	91	17	9		0
	*S. macrocarpum*	150	67	73	33	9		18
*T. recurvata*	*B. bipinnata*	150	92	37	1	47	7	16
	*B. copalloifera*	150	98	45	2	28		27
	*B. fagaroides*	150	59	29	21	13	20	58
	*I. murucoides*	150	67	52	33	13		35
	*I. pauciflora*	150	88	34	12	26		40
	*S. macrocarpum*	150	99	38	1	34		28
*T. schiedeana*	*B. bipinnata*	150	92	77	8	23		0
	*B. copalloifera*	150	71	47	29	21		33
	*B. fagaroides*	150	88	69	12	9		21
	*I. murucoides*	150	90	61	10	21		18
	*I. pauciflora*	150	75	89	25	8		3
	*S. macrocarpum*	150	57	68	43	20		12

The seeds used were viable and germinated *in situ* (Table 3); the percentage of germination in the controlled treatments was maintained above 50 % and sometimes reached 100 %. Germination of the seeds in the lots exposed to herbivores differed from that of the control lots (Table 3), but this effect was caused by the seed removal, since less seeds were available to germinate in the lots where removal occurred.

**Figure 1.**
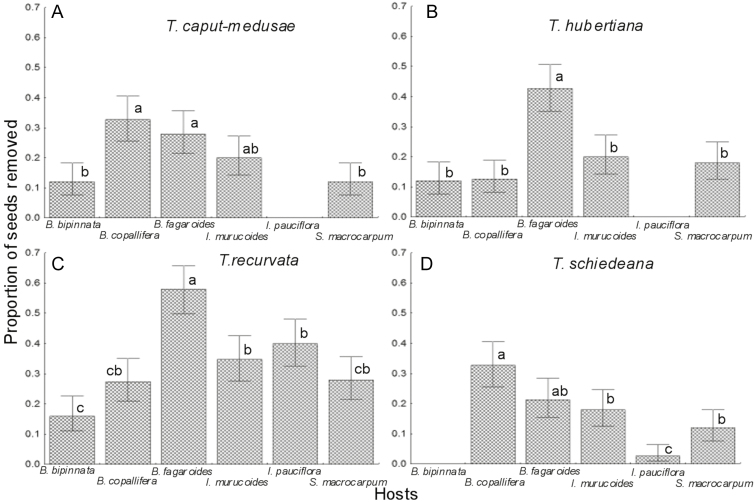
Proportion of seeds depredated in four species of epiphytes placed on six host tree species of the tropical dry forest of San Andrés de la Cal, Tepoztlán, in Morelos, Mexico. Different letters denote significant differences in seed depredation among host species. The lines of dispersion are binomial 95 % confidence intervals.

For the four species of epiphytes (*T. caput-medusae*, χ^2^ = 31.90, *P* < 0.0001; *T. hubertiana*, χ^2^ = 51.9, *P* < 0.0001; *T. recurvata*, χ^2^ = 68.51, *P* < 0.0001 and *T. schiedeana*, χ^2^ = 58.3, *P* < 0.0001; Fig. 1), there were differential removal rates of seeds among the host species. In the case of *T. caput-medusae* (Fig. 1A), the hosts that presented the highest removal of seeds were *B. copallifera* and *B. fagaroides*, while no seed removal occurred in *I. pauciflora*. For the epiphyte *T. hubertiana* (Fig. 1B), we found a high removal of seeds in *B. fagaroides*, while no seeds were removed in *I. pauciflora*. In the *T. recurvata* lots (Fig. 1C), removal of seeds was higher in *B. fagaroides* and lower in *B. bipinnata*. In *T. schiedeana* (Fig. 1D), removal of seeds was higher in *B. copallifera* and lower in *I. pauciflora*, while no seeds were removed from *B. bipinnata*.

**Figure 2.**
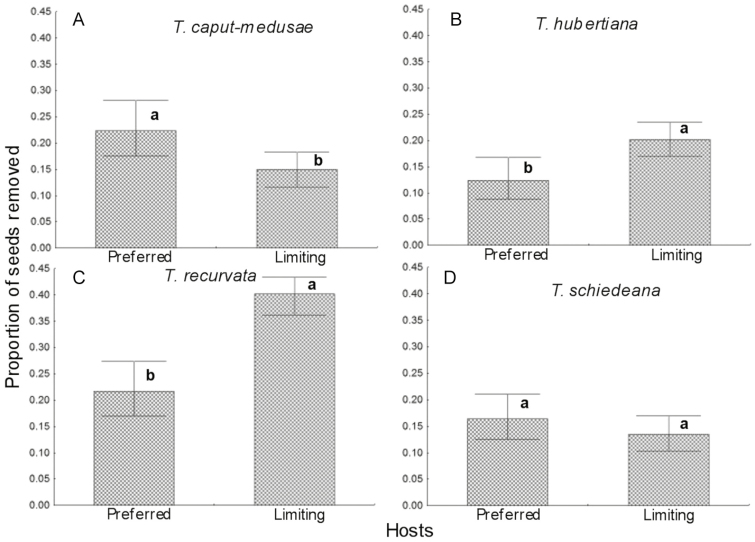
Proportion of removed seeds of four epiphyte species grouped into preferred (*Bursera copallifera*, *B. bipinnata*) and limiting (*B. fagaroides*, *Ipomoea murucoides* and *I. pauciflora*) host species. Different letters denote significant differences in seed depredation among host species. The lines of dispersion are binomial 95 % confidence intervals.

**Figure 3.**
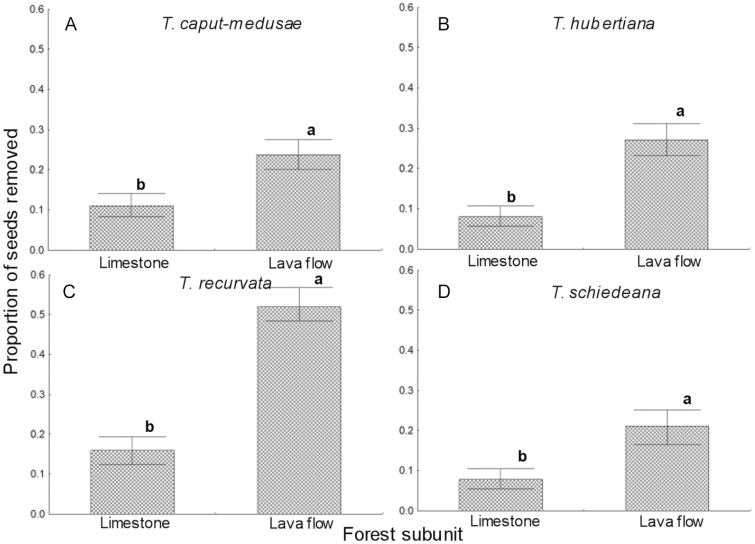
Proportion of seeds of four epiphyte species removed, according to the forest subunit (soils developed on limestone or on lava flow) where the six host tree species were selected in the tropical dry forest of San Andrés de la Cal, Tepoztlán, in Morelos, Mexico. Different letters denote significant differences in seed depredation among host species. The lines of dispersion are binomial 95 % confidence intervals.

On comparing the extent to which the ants removed the seeds from trees classed as preferred or limiting hosts (Fig. 2), we found that there was a differential removal of seeds in three epiphyte species between the two groups of hosts (*T. caput-medusae*, χ^2^ = 5.70, *P* = 0.0169; *T. hubertiana*, χ^2^ = 18.55, *P* < 0.00001; and *T. recurvata*, χ^2^ = 13.86, *P* < 0.0001), while removal of *T. schiedeana* seeds (Fig. 3D) did not differ between the groups of hosts (χ^2^ = 2.49, *P* = 0.1143). For *T. caput-medusae* (Fig. 3A), the highest removal occurred in the preferred hosts, while for *T. hubertiana* and *T. recurvata* the highest removal occurred in the limiting hosts (Fig. 2B and C).

On comparing seed removal between the two forest subunits (limestone and lava flow) in which the host trees were located, we found differences in the seeds of *T. caput-medusae* (χ^2^ = 26.22, *P* < 0.0001), *T. hubertiana* (χ^2^ = 66.67, *P* < 0.00001), *T. schiedeana* (χ^2^ = 50.79, *P* < 0.0001) and *T. recurvata* (χ^2^ = 54.49, *P* < 0.00001). Removal was consistently higher in the lava-flow tropical dry forest subunit compared to limestone subunit (Fig. 3).

**Figure 4.**
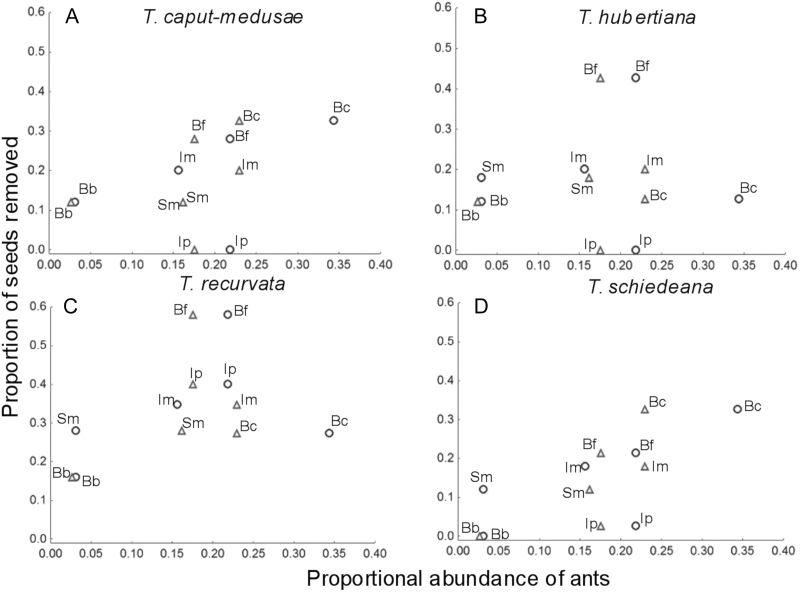
Relationship between the proportion of removed seeds of four epiphyte species and the abundance of two ant species (*Camponotus rectangularis willowsi*, circles; *Crematogaster curvispinosa*, triangles) on six host tree species of the tropical dry forest of San Andrés de la Cal, Tepoztlán, in Morelos, Mexico. Bb = *Bursera bibinnata*; Bc = *B. copallifera*; Bf = *B. fagaroides*; Im = *Ipomoea murucoides*; Ip = *I. pauciflora*; Sm = *Sapium macrocarpum*.

We found a significant correlation between the mean proportion of removed seeds and ant abundance (Spearman correlation, *r*_s_ = 0.71, *P* < 0.05) (Fig. 4). When removal of seeds of the four epiphyte species was related to the abundance of the ant species *C. r. willowsi* and *C. curvispinosa*, the most consistent pattern was the lower the ant abundances the lower the seed removal (Fig. 4). For seeds of the epiphyte *T. caput-medusae*, we found both a greater removal and a higher abundance of ants in *B. copallifera* compared to the other tree species (Fig. 4A). For seeds of *T. hubertiana* (Fig. 4B) and *T. recurvata* (Fig. 4C), we found a high removal of seeds and an intermediate abundance of ants in *B. fagaroides*. For *T. schiedeana* (Fig. 4D), the tree with the highest removal of seeds was *B. copallifera*. This tree species also presented the highest abundance of ants.

Removal of seeds increased the number of cases of failure to establish, since the removal could have been greater than the number of seeds that did not germinate. In all of the *Tillandsia* species the highest ratio of removed seeds:non-germinated seeds occurred in the host *B. fagaroides* (Table 3), ranging from 2.3:1 for the epiphyte *T. schiedeana* to 8.6:1 for the epiphyte *T. hubertiana*.

## Discussion

Tree-insect coexistence has favoured the evolution of associations, such as the ant-tree association, and it has been hypothesized that this association could explain the absence or low abundance of structural parasites on trees (Janzen 1971) since the ants, while defending the trees, remove the seeds of structural parasites. In the study area, we do not know whether the ants defend the species of trees studied, but they do forage in these trees and use resources, such as nectar or pollen, which are available during the period of dispersion, germination and establishment of the seeds of the *Tillandsia* species studied. This study tested experimentally whether the ants differentially remove seeds of epiphyte plants that were placed on trees with different epiphyte abundances. The evidence shows that the ants effectively remove the seeds of *T. caput-medusae*, *T. hubertiana*, *T. schiedeana* and *T. recurvata*. In all of the trees, ants were both observed and found trapped in the tanglefoot barrier, but removal activity was found to differ with the particular *Tillandsia* species, as well as the tree species and forest subunit.

In other studies, the germination of *Tillandsia* seeds reported under natural conditions has been <30 % (Bernal *et al.* 2005; Mondragón and Calvo-Irabien 2006; Toledo-Aceves and Wolf 2008; Toledo-Aceves *et al.* 2012); however, we observed a high germination (>50–100 %), showing that viable seeds were being provided to the herbivores. The data also indicate that non-flying organisms conduct the seed removal, since there was no removal in the lots protected by a tanglefoot barrier and where there were no bridges. As suggested in other studies (Youngsteadt *et al.* 2009), the data indicate that ants constitute the main seed removers.

For the epiphyte species *T. recurvata*, the removal pattern complies with the hypothesis (i.e. the activity of seed removal by ants in tree species with lower epiphyte loads was greater than that in trees with higher epiphyte loads); greater seed removal was found in the limiting hosts *B. fagaroides*, *I. pauciflora* and *I. murucoides*, compared with the preferred hosts *B. bipinnata* and *B. copallifera*. These latter host species presented low seed removal while, in *S. macrocarpum* (limiting), seed removal was intermediate. Removal of seeds of the species *T. recurvata* and *T. hubertiana* was high and the highest removal occurred in the limiting host *B. fagaroides*, which in all cases presented an intermediate level of ant activity. This indicates that, for this host, seed removal is more important than low seed germination.

For the seeds of *T. caput-medusae* and *T. schiedeana*, a constant pattern of very low or zero seed removal was found in *I. pauciflora*, despite ant activity and a germination greater than 70 % being recorded, demonstrating the viability of these seeds. Some tree species (*C. multiflora*, *I. murucoides* and *S. macrocarpum*) are considered limiting for epiphytes because they present allelopathic substances in the bark that can act to inhibit seed germination (Valencia-Díaz *et al.* 2010; Fernández-García 2014). In *I. murucoides*, *I. pauciflora* and *S. macrocarpum*, we found high seed germination, low seed removal and low inhibition of germination. The high germination found in these limiting hosts contradicts previous evidence that shows that their bark contain substances that inhibit germination (Valencia-Díaz *et al.* 2010); however, this high germination could have occurred because the seeds were placed on the tree branches once the rainy season had started. This suggests that the rains serve to wash the allelopathic substances from the bark (i.e. the seed germination of seeds will be greater during the rainy season, in comparison with the dry season; Victoriano-Romero 2013).

On the preferred host *B. copallifera*, we found high levels of ant activity and a high removal of seeds of *T. caput-medusae* and *T. schiedeana*. Seasonal patterns in the floral resources may determine the structure of the visitors, influencing their foraging behaviour (Kremen *et al.* 2007). During the experiment, *B. copallifera* flowered (May–June) and fructified (July–September) (Rzedowski *et al.* 2004). This suggests that the high removal could be caused by resource defence behaviour exhibited by the ants. It has been documented that some ant species of the genera *Camponotus* and *Crematogaster* use floral or extrafloral nectar, and it is possible that the instinct of the ants to guard the nectar of the *B. copallifera* flowers has thus caused the increased seed removal (Oliveira and Brandao 1991; Díaz-Castelazo *et al.* 2004). Testing this hypothesis would require further study.

Trees of *B. copallifera* present high abundances of *Tillandsia* (Vergara-Torres *et al.* 2010; Cortés-Anzúres 2015) and it is possible that this high abundance of epiphytes could counteract the effect of removal. The high abundance of epiphytes implies that many fruits may be produced and the resinous branches of *B. copallifera* could capture up to half of the seeds that are produced by the epiphytic plants it hosts (Victoriano-Romero *et al.* 2017). The high abundance of epiphytes and high capture of seeds in *B. copallifera* means that it is possible that some seeds avoid removal and germinate since the bark of this species is not phytotoxic (Valencia-Díaz *et al.* 2010).

The data show that the particular forest subunit influences seed removal, probably because of differences in the ant fauna among subunits. It has been documented that ants of the genus *Crematogaster* present monopolistic behaviour (i.e. aggressive defence of its resources; Eskildsen *et al.* 2001). In the lava-flow tropical dry forest subunit, *C. curvispinosa* is the most abundant ant species but is absent from the limestone-rock tropical dry forest. The higher seed removal in the lava-flow tropical dry forest compared to the other subunit may be due to *C. curvispinosa* being a more granivorous ant species than *C. r. willowsi*, the latter being found in both tropical dry forest subunits. Although in the forest subunit where they coexist, the ant species develop competitive behaviour that has repercussions in the form of increased *Tillandsia* seed removal.

In this study, we have shown that ant depredation of *Tillandsia* seeds can cause a significant reduction in the quantity of seeds available for germination. Similar results have been found in other plant species, where ants have reduced establishment by up to 50 % (Setterfield and Andersen 2018). In the canopy, other factors influence epiphyte community structure; for example, it is possible that, in some hosts, high rates of depredation may not have a visible impact on recruitment; if seed production is high, the seeds can be captured by the host itself (Victoriano-Romero *et al.* 2017), satiating the ant community.

Interaction with the bark is of great importance for germination of epiphyte seeds (Valencia-Díaz *et al.* 2010); however, we found that seed removal could be equally or more important than the lack of germination. Evidence suggests that *Tillandsia* seeds can germinate on any inert substrate (Abril and Bucher 2009; Wester and Zotz 2010; Victoriano-Romero 2013) and the results of this study support this notion. However, some host species capture few *Tillandsia* seeds (Victoriano-Romero *et al.* 2017) and have allelopathic compounds that reduce seed germination (Valencia-Díaz *et al.* 2010). In these host species, high seed depredation by ants will more severely reduce seedling establishment.

Some studies have described mutualist defensive associations between ants and plants (Janzen 1966, 1967; Huxley and Cutler 1991; Rico-Gray and Oliveira 2007), in which the ants protect the plants from attack by herbivores or invasion by structural parasites (e.g. epiphytes). We did not identify all the mechanisms of association between the ants and the tree species, or even with the epiphyte species themselves, but our results clearly suggest that seed depredation by ants reduces the amount of seed of epiphytes available for establishment. Exploring the mechanisms of association between trees and ants will help us to better understand the structure of the epiphytic plant community. The ants are key interactors that, with their patrolling and resource (flowers, nectar, domatia) defence activities, reduce the abundance of epiphytic plants on the trees. Further experiments are necessary to further our understanding about the tree resources that are defended by ants, which trigger ant defensive behaviour, causing a reduction in the number of epiphyte seeds available for establishment.

## Conclusions

We conclude that in the studied tropical dry forest canopy, ants reduce the number of *Tillandsia* seeds available for germination. This ant behaviour could be more important that failures in seed germination and depends on both the host species where seeds are available and the forest subunit. This variation could be due to different host resources and the presence of competing ants.

## Contributions by the Authors

All authors contibuted equally to this manuscript.

## Conflict of Interest

None declared.
